# Oral Contraceptives after Myomectomy: A Short Term Trial

**DOI:** 10.1155/2009/476897

**Published:** 2009-07-28

**Authors:** Stefano Luisi, Valentina Ciani, Massimo Gabbanini, Sofia Sollazzi, Michela Torricelli, Francesco Calonaci, Felice Petraglia

**Affiliations:** Obstetrics and Gynecology Unit, Department of Pediatrics, Gynecology, and Reproductive Medicine, University of Siena, 53100 Siena, Italy

## Abstract

Following myomectomy the rate of fertility is restored and pregnancy may be attempted with a good outcome. In the present study a 3 month treatment with OCs in a group of women after a myomectomy was evaluated. The drug compliance and side effects, the benefits of OC in order to reduce symptoms, to increase post-surgical hemoglobin levels and to avoid an early pregnancy after myomectomy were analyzed. A group of women (*n* = 55) each with myoma ≥5 cm was recruited: they presented menorrhagia, pelvic pain, dyspareunia and dysmenorrhae. After laparotomic myomectomy the women were divided into 3 groups. Group 1: women (*n* = 16) treated with pill A (15 mcg of ethynilestradiol + 60 mcg of gestodene); group 2: women (*n* = 23) treated with pill B (20 mcg of ethynilestradiol + 100 mcg of levonorgestrel); group 3: women (*n* = 16) treated with a placebo (oral calcium). After three months from myomectomy and treatment patients in each group reported a reduced menorrhagia, dismenorrhea and pelvic pain. Serum haemoglobin levels increased in all women (*P* < .05). No pregnancy occurred in any group and the compliance was good. A post surgery treatment by using oral contraceptives guarentees pregnancy prevention, associated with reduction of pain, and improvement of haematologic conditions.

## 1. Introduction

Uterine leiomyomata is a major source of morbidity among women of reproductive age [1]. They are benign tumors of smooth muscle, commonly referred to as fibroids. Despite the importance of these tumors, little is still known about their epidemiology or aetiology [2]. Epidemiologic studies suggest that risk is inversely associated with age at menarche, parity and age at first birth and positively associated with years since last term birth [3], so the incidence of leiomyomata rises through out the reproductive years [2, 4], while the incidence of surgery is lowest in the menopausal years [4, 5]. This pattern suggests a dependence on ovarian steroid hormones, but the role of these agents in the etiology of uterine leiomyomata remains unclear [6, 7]. Uterine fibroids cause a variety of symptoms, such as menorrhagia, pelvic pain, infertility, reproductive dysfunction (like pregnancy loss and pregnancy complications), but sometimes they are completely asymptomatic [8]. The effect of estrogen-progestin oral contraceptives (OCs) on the volume of uterine leiomyomata is not well characterized. 

 OCs may play a role in the development or growth of leiomyomata [9] and a significantly elevated risk among women who first used OCs at ages 13–16 years compared with non-OCs users has been shown [1]. On the other hand, the little available epidemiologic data have suggested a protective effect of OCs in the risk of fibroids [10, 11]. Low-dose OC use provides the benefit of a reduction in the duration of menstrual bleeding, with resultant improvement in haemoglobin levels, without increasing uterine size [12]. Antiprogesterone which induces ovarian acyclicity also decreases size of leiomyomata, thus antiprogesterone may provide a novel mode of management of leiomyomata [13]. 

 Therefore, the association between oral contraceptive and the risk of uterine fibroids is still unclear. Uterine leiomyomata is the fifth cause of hospitalization for gynecologic conditions unrelated to pregnancy in women aged 15–44 and the primary indications for hysterectomy among women of all ages [14]. The range of alternatives to hysterectomy includes medical regimens (levonorgestrel- releasing intrauterine system), a wide range of endometrial ablative techniques, and where fibroids are the primary pathology myomectomy and uterine artery embolization. 

 Abdominal myomectomy is an effective surgical alternative to hysterectomy one for therapy of symptomatic uterine fibroids, it preserves fertility and femininity [15]. Myomectomy is associated with a favourable outcome in infertile women, particularly if no other complication variable is present [16]. The location of the myomata may play an important role in determining infertility. Both large intramural and subserous myoma are thought to interfere with conception and reduce the effectiveness of assisted reproduction cycles [17]. The size of the myoma may represent another important prognostic factor, 5 cm in diameter being the size limit which appears to justify myomectomy. Restoration of fertility after myomectomy has been reported, with pregnancy rates ranging between 44 and 52% [18]. 

 The time to postmyomectomy conception is short, with ~80% of pregnancies occurring during the first year following surgery, however, in the first months after a myomectomy a gestation the risk of uterine rupture is higher [17]. 

 In the present study a 3-month treatment with OCs in a group of women after a myomectomy was evaluated. In addition, the drug compliance and side effects, the benefits of OC in order to reduce symptoms, to increase post-surgical hemoglobin levels and to avoid an early pregnancy after myomectomy was analyzed. Moreover, we investigated the risk of uterine leiomyomata in relation to short-term contraceptive use.

## 2. Materials and Methods

The study was approved by the Institutional Review Board of the Academic Health Center of Siena, and an informed consent was obtained from each participant. 

 A group of 55 women (aged 30–45, BMI 22–25) with ultrasound and a histologically confirmed diagnosis of uterine fibroids was recruited from September 2006 to April 2007. All the women presented unexplained infertility and had only one large intramural or subserosal myoma measuring ≥5 cm and underwent on laparotomic myomectomy (these myomas were not as suitable for the laparoscopic approach). None of the subjects had taken any medications for at least 3-months before the study. Most of them presented a variety of female reproductive problems and symptoms, such as menorrhagia (excessive uterine bleeding occurring at regular intervals or prolonged uterine bleeding lasting more than seven days) [19] or irregular bleeding (*n* = 36), pelvic pain (*n* = 22), dyspareunia and dysmenorrhoea (*n* = 15). 

 To investigate the outcome, women were instructed to keep (i) a diary of menstrual bleeding (number of days of menstrual flow and total number of pads/tampons used), rating the blood loss on a visual analog scale from zero (no blood loss) to 10 (gushing-type bleeding); (ii) the presence of side effects and overall satisfaction with treatment was rated on a five-level scale (very satisfied, satisfied, uncertain, dissatisfied, and very dissatisfied); (iii) a questionnaire for the assessment of pain symptoms (dysmenorrhea, dyspareunia and pelvic pain) by using a 10 points Visual Analog Scale (VAS) [20]. 

 The clinical characteristics of the women enrolled in the study are shown in Table 1, while the characteristics of the myomas are shown in Table 2. 

 Exclusion criteria were von Willebrand's disease or coagulopathies (known or suspected), or a history of hormone-dependent malignancies; known or a history of deep-vein thrombosis, active thrombophlebitis, thromboembolic disorder, or cerebrovascular accident, myocardial infarction or ischemic heart disease, untreated hypertension, liver disease, any endocrine disorder other than controlled thyroid disease, and a smoking habit of one pack or more of cigarettes per day. 

 After laparotomic myomectomy and a normal postoperation time (5 days) without post-operative complications, the women were divided into three randomized groups according to the treatment:

- group 1: treated with pill A (*n* = 16) (15 mcg of ethynilestradiol + 60 mcg of gestodene) (Arianna, Bayer Schering, Berlin, Germany);
- group 2: treated with pill B (*n* = 23) (20 mcg of ethynilestradiol + 100 mcg of levonorgestrel) (Miranova, Bayer Schering, Berlin, Germany);
- group 3: treated with placebo (*n* = 16) (oral calcium).

We used two different pill formulations to compare if there were any differences between second (levonorgestrel) and third (gestodene) generation progestin. 

 Since it was a blind study to each group was said to use barrier contraception in order to avoid pregnancy. All the women were controlled in the following 3-months after surgery. 

 Before and after 3-months a pelvic transvaginal ultrasound (LAB 70 ESAOTE SpA, Genova, Italy) evaluation was done, and peripheral blood was drawn to measure red and white blood cell count, hematocrit, hemoglobin, and platelets. 

 The serum results are expressed as a mean of ±SE, and differences between groups were assessed by using the unpaired *t*-test. Probability values of less than .05 were considered statistically significant.

## 3. Results

**Figure 1:**
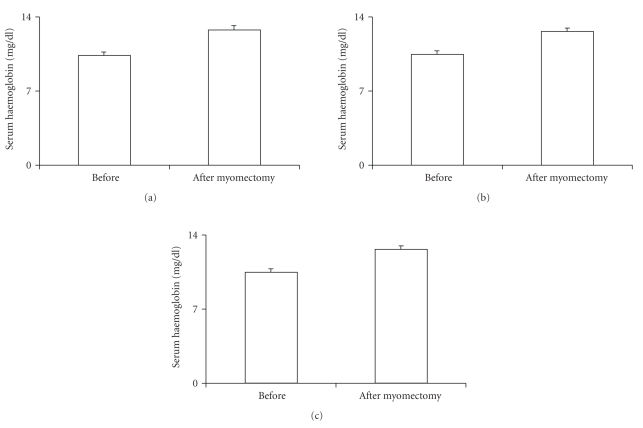
Serum haemoglobin concentration after treatment in the three groups. Group 1: women treated with 15 mcg of ethynilestradiol + 60 mcg of gestodene; group 2: women treated with 20 mcg of ethynilestradiol + 100 mcg of levonorgestrel; group 3: women treated with a placebo. * = *P* < .05.

After the treatment the three groups of patients showed reduced menorrhagia, dysmenorrhoea, pelvic pain (*P* < .01) and increased serum haemoglobin concentration (*P* < .05) (Figure 1). The haemoglobin levels showed to be about the same in the three groups after myomectomy. 

**Figure 2:**
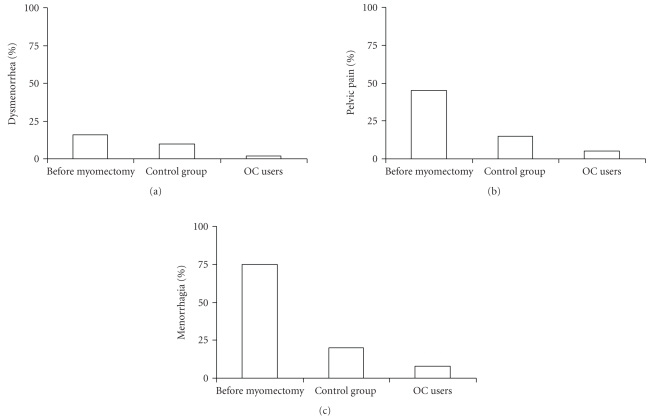
Improvement of symptoms (dysmenorrhoea, pelvic pain, menorrhagia) after myomectomy and OCs use.

 During the transvaginal pelvic ultrasound no gynecological complications were noted in any of the subjects. A reduction of symptoms occurred in OCs users faster than in the control group (Figure 2). No pregnancy was reported in any of the patients. 

 No relevant side effects (spotting, bloating, acne, mood swing, weight gain or loss, headaches, breast pain) were registered in any of the groups after 3-months of treatment.

## 4. Discussion

The present results showed that a 3-month treatment with OCs after myomectomy is associated with regular menstrual cycles, increased serum haemoglobin concentration without pelvic pain, dysmenorrhoea, and risk of pregnancy. 

 Myomectomy is frequently performed to preserve or increase fertility, although the risk of future uterine rupture is a major concern of any surgery to the uterus. For this reason it is necessary to avoid pregnancy in the first months after the surgery when the risk is higher [21]. Women recruited in our present study were >30 years and each had a intramural or subserosal myoma. The rationale of the OC treatment was to give a contraceptive cover at least in the first 3-months after myomectomy. 

 In women with menstrual disorders, such as menorrhagia, the OC treatment reduces the menstrual blood loss and increases the serum haemoglobin concentration, reducing the incidence of anemia. ﻿Oral contraceptives represent also the main medication used to treat dysmenorrhea. Although several mechanisms underlying the dysmenorrheal pain relief attained by OC use have been reported, suppression of prostaglandin (PG) synthesis, which leads to reduced uterine contractions, represents one of the most probable pathways [22]. 

 The effect of OCs on myomas is still not fully clear. Evidence showed that both reproductive factors and oral contraceptive use at a young age, influence the risk of uterine leiomyomata among premenopausal women [23]. In a case report, a 45-year-old woman, with a symptomatic uterine leiomyoma, presented a reduced myoma volume after the discontinuation of OCs [24]. 

 To the contrary after a 1-year study Friedman et al. showed that in most women with leiomyomas, low-dose OC use provides the noncontraceptive benefit of a reduction in the duration of menstrual flow, with resultant improvement in hematocrit, without increasing uterine size [25]. 

 The prolonged use of the last generation of oral contraceptives does not increase the uterine myoma volume and furthermore it produces a noteworthy reduction in the duration of menstrual flow with consequent increase in hematocrit [26]. 

 We chose a short-term trial since the majority of women undergoing myomectomy despite of hysterectomy for fibroids want a pregnancy in the immediate postoperative period. We suggested them the use of the pill to improve symptoms and menstrual bleeding. Moreover, we wanted to underlined the rapid improvement of the clinical condition during the first 3-months after surgery in association with oral contraceptive. 

 Therefore, an oral contraceptive post-surgery treatment for at least 3-months guarantees the prevention of pregnancy, allowing a faster improvement of the clinical and haematologic conditions, without increasing the risk of recurrency.

**Condensation**

The use of a low-dose of estro-progestins after myomectomy guarantees the prevention of pregnancy, allowing a faster improvement of the clinical and haematologic conditions.

## Figures and Tables

**Table 1:** Distribution of patients according to age, dysmenorrhoea, menometrorrhagia and pelvic pain at previous surgery.

Variable	Patients (*n *°)	%
Age		
* *30–35 years	9	16
* *35–40 years	42	77
* * >40 years	4	7

Presence of symptoms	48	87
* *Dysmenorrhoea	8	16
* *Menometrorrhagia	36	75
* *Pelvic pain	22	45

Absence of symptoms	7	13

**Table 2:** Distribution of myomas according to location and diameter.

Myoma	Patients	%
Diameter (cm)		
* *5 − < 8	22	40
* *8 − < 10	27	49
* * >10	6	11

Location		
* *Intramural	42	76
* *Subserous	13	24

## References

[1] Marshall LM, Spiegelman D, Goldman MB (1998). A prospective study of reproductive factors and oral contraceptive use in relation to the risk of uterine leiomyomata. *Fertility and Sterility*.

[2] Ross RK, Pike MC, Vessey MP,  Bull D, Yeates D,  Casagrande JT (1986). Risk factors for uterine fibroids: reduced risk associated with oral contraceptives. *British Medical Journal*.

[3] Parazzini F, La Vecchia C, Negri E, Cecchetti G, Fedele L (1988). Epidemiologic characteristics of women with uterine fibroids: a case-control study. *Obstetrics and Gynecology*.

[4] Marshall LM, Spiegelman D, Barbieri RL (1997). Variation in the incidence of uterine leiomyoma among premenopausal women by age and race. *Obstetrics and Gynecology*.

[5] Romieu I, Walker AM, Jick S (1991). Determinants of uterine fibroids. *Post Marketing Surveillance*.

[6] Yantiss RK, Clement PB, Young RH (2000). Neoplastic and pre-neoplastic changes in gastrointestinal endometriosis: a study of 17 cases. *American Journal of Surgical Pathology*.

[7] Lumsden MA, Wallace EM (1998). Clinical presentation of uterine fibroids. *Bailliere's Clinical Obstetrics and Gynaecology*.

[8] Andersen J, Barbieri RL (1995). Abnormal gene expression in uterine leiomyomas. *Journal of the Society for Gynecologic Investigation*.

[9] John AH, Martin R (1971). Growth of leiomyomata with estrogen—progestogen therapy. *Journal of Reproductive Medicine for the Obstetrician and Gynecologist*.

[10] Parazzini F, Negri E, La Vecchia C, Fedele L, Rabaiotti M, Luchini L (1992). Oral contraceptive use and risk of uterine fibroids. *Obstetrics and Gynecology*.

[11] Ratner H (1986). Risk factors for uterine fibroids: reduced risk associated with oral contraceptives. *British Medical Journal*.

[12] Friedman AJ, Thomas PP (1995). Does low-dose combination oral contraceptive use affect uterine size or menstrual flow in premenopausal women with leiomyomas?. *Obstetrics and Gynecology*.

[13] Murphy AA, Morales AJ, Kettel LM, Yen SSC (1995). Regression of uterine leiomyomata to the antiprogesterone RU486: dose-response effect. *Fertility and Sterility*.

[14] Farquhar CM, Steiner CA (2002). Hysterectomy rates in the United States 1990–1997. *Obstetrics and Gynecology*.

[15] Banu NS, Manyonda IT (2005). Alternative medical and surgical options to hysterectomy. *Best Practice and Research: Clinical Obstetrics and Gynaecology*.

[16] Connolly G, Doyle M, Barrett T, Byrne P, De Mello M, Harrison RF (2000). Fertility after abdominal myomectomy. *Journal of Obstetrics and Gynaecology*.

[17] Stovall DW, Parrish SB, Van Voorhis BJ, Hahn SJ, Sparks AET, Syrop CH (1998). Uterine leiomyomas reduce the efficacy of assisted reproduction cycles: results of a matched follow-up study. *Human Reproduction*.

[18] Chapron C, Dubuisson J-B (1996). Laparoscopic treatment of deep endometriosis located on the uterosacral ligaments. *Human Reproduction*.

[19] Apgar BS, Kaufman AH, George-Nwogu U, Kittendorf A (2007). Treatment of menorrhagia. *American Family Physician*.

[20] Larroy C (2002). Comparing visual-analog and numeric scales for assessing menstrual pain. *Behavioral Medicine*.

[21] Lieng M, Istre O, Langebrekke A (2004). Uterine rupture after laparoscopic myomectomy. *Journal of the American Association of Gynecologic Laparoscopists*.

[22] Davis AR, Westhoff C, O'Connell K, Gallagher N (2005). Oral contraceptives for dysmenorrhea in adolescent girls: a randomized trial. *Obstetrics and Gynecology*.

[23] Marshall LM, Spiegelman D, Goldman MB (1998). A prospective study of reproductive factors and oral contraceptive use in relation to the risk of uterine leiomyomata. *Fertility and Sterility*.

[24] Barbieri RL (1997). Reduction in the size of a uterine leiomyoma following discontinuation of an estrogen-progestin contraceptive. *Gynecologic and Obstetric Investigation*.

[25] Friedman AJ, Thomas PP (1995). Does low-dose combination oral contraceptive use affect uterine size or menstrual flow in premenopausal women with leiomyomas?. *Obstetrics and Gynecology*.

[26] Larsson G, Milsom I, Lindstedt G, Rybo G (1992). The influence of a low-dose combined oral contraceptive on menstrual blood loss and iron status. *Contraception*.

